# Prevalence and Control of Hypertension and Albuminuria in South Korea: Focus on Obesity and Abdominal Obesity in the Korean National Health and Nutrition Examination Survey, 2011–2012

**DOI:** 10.1371/journal.pone.0111179

**Published:** 2014-10-31

**Authors:** Su-Jung Yoon, Do-Hoon Kim, Ga-Eun Nam, Yeo-Joon Yoon, Kyung-Do Han, Dong-Wook Jung, Sang-Woon Park, Young-Eun Kim, Sung-Ho Lee, Sang-Su Lee, Yang-Hyun Kim

**Affiliations:** 1 Department of Family Medicine, Korea University College of Medicine, Seoul, South Korea; 2 Department of Medical Statistics, Catholic University College of Medicine, Seoul, South Korea; Tulane School of Public Health and Tropical Medicine, United States of America

## Abstract

**Background:**

Albuminuria is associated with cardiovascular disease, and the relationship between albuminuria and hypertension is well established in many studies. So the control of hypertension is critical for decreasing cardiovascular events and albuminuria. Obesity and abdominal obesity are also associated with hypertension and albuminuria. Therefore, we analyzed the relationship between albuminuria and the prevalence and control of hypertension in the general Korean population according to obesity status.

**Methods:**

We analyzed data from the 2011–2012 Korea National Health and Nutrition Examination Survey, and 9,519 subjects were included. Subjects were divided into four groups: non-obese/normal waist circumference, non-obese/high waist circumference, obese/normal waist circumference, and obese/high waist circumference.

**Results:**

Systolic blood pressure and diastolic blood pressure were positively associated with albumin–creatinine ratio in all groups (all p values <0.005). Non-obese/normal waist circumference group were more likely to have hypertension (odds ratios [95% confidential intervals (CIs)] were 3.20 [2.21–4.63] in microalbuminuria level and 3.09 [1.05–9.14] in macroalbuminuria level), and less likely to have controlled hypertension (odds ratios <1 for both albuminuria levels) after adjusting for all covariates. Obese/normal waist circumference group were also more likely to have hypertension (odds ratio [95% CI] were 3.10 [1.56–6.15] in microalbuminuria level and 21.75 [3.66–129.04] in macroalbuminuria level), and less likely to have controlled hypertension in macroalbuminuria level (odds ratio [95% CI], 0.04 [0.01–0.15]).

**Conclusions:**

Non-obese and normal waist circumference subjects have an increased prevalence and decreased control of hypertension in microalbuminuria and macroalbuminuria levels. Screening for albuminuria may provide helpful information about hypertension and blood pressure control, particularly in the non-obese and normal waist circumference subjects.

## Introduction

The prevalence of cardiovascular disease (CVD) is gradually increasing in many countries, and the treatment and management of CVD represent a significant cost burden to society. Therefore, many countries are working to reduce the impact of cardiovascular risk factors such as hypertension (HTN), diabetes mellitus (DM), dyslipidemia, and obesity [1]–[3]. Albuminuria, which is associated with diabetic nephropathy, is a risk factor for CVD [4]–[6]. It is conventionally diagnosed when the urine albumin–creatinine ratio (ACR) is ≥30 mg/g [7], [8]. Epidemiologic studies have shown that excess urinary albumin excretion is related to the increased risk of CVD and associated with increased cardiovascular and all-cause mortality [6], [9]–[13].

Among the aforementioned cardiovascular risk factors, HTN has shown a positive correlation with albuminuria in many studies [14]–[19]. This relationship has been generally explained by vascular impairment such as endothelial dysfunction [20] and direct transmission of elevated pressure to the glomerulus in the kidney [21], [22]. Therefore, uncontrolled HTN, defined as blood pressure ≥140/90 mmHg, may increase the prevalence of CVD and progression of renal disease [23],[24]. However, controlled HTN is associated with low CVD death rate and decreased prevalence of complications such as myocardial infarction, stroke, and microalbuminuria [25], [26]. Studies conducted in Korea also have investigated the relationship between albuminuria and HTN, but only included community-based samples targeting type 2 DM subjects or the general population [27]–[30]. In addition to the association with HTN, microalbuminuria is also associated with obesity and abdominal obesity, which are generally associated with HTN [31]–[36]. Therefore, we analyzed the relationship between the prevalence and control of HTN and albuminuria in the general South Korean population according to the obesity status (including obesity and abdominal obesity).

## Materials and Methods

### Survey overview

We analyzed data from the 2011–2012 Korean National Health and Nutrition Examination Survey (KNHANES). The Division of Chronic Disease Surveillance at the Korean Center for Disease Control and Prevention (KCDC) initiated the KNHANES in 1998 as a nationwide survey. The KNHANES is designed to evaluate the national health and nutrition level, and consists of a physical exam, health interview, and nutritional evaluation [37]. To obtain a representative sample of non-institutionalized civilians of both sexes from all geographic regions of South Korea, the subjects of this study were prorated by age from the 2005 census registry, stratified in multiple stages, and selected using a cluster sampling design.

### Subjects

A total of 12,859 participants were aged ≥19 years in the KNHANES. We excluded subjects with liver cirrhosis, chronic hepatitis B or C, pulmonary and extrapulmonary tuberculosis, and cancer, as well as those who were pregnant. Subjects who did not answer questions about medical history and those who had not fasted for >8 h before blood sample collection were also excluded. Finally, 9,519 subjects were included in this study. All participants provided written informed consent, and the study protocol was approved by the institutional review board of the KCDC.

### Lifestyle and sociodemographic variables

Smoking status, alcohol consumption, and physical activity were assessed via a self-reported questionnaire. Current smokers were defined as those who currently smoked and had smoked ≥100 cigarettes in their lifetime. For alcohol consumption, subjects who drank ≥30 g/day were classified as heavy drinkers. Physical activity was defined using the International Physical Activity Questionnaire (IPAQ) [38]. The regular exercise group included subjects who exercised moderately more than five times a week for >30 min per session or those who exercised intensively more than three times a week for >20 min per session. Household income was adjusted for the number of family members, and we divided the household income into quartiles. We defined ‘low income’ as the lowest quartile of income, and ‘low education’ as a duration of education of ≤9 yrs.

### Anthropometric and laboratory measurements

Skilled staff members performed physical examinations according to standard procedures. Subjects wore light clothing without shoes during weight and height measurements, which were measured to the nearest 0.1 kg and 0.1 cm, respectively. Waist circumference (WC) was measured at the narrowest point between the lower rib margin and iliac crest during exhalation. Systolic blood pressure (SBP) and diastolic blood pressure (DBP) were measured three times at 5 min intervals using a standard mercury sphygmomanometer (Baumanometer, WA Baum Co. Inc., Copiague, NY, USA), and the mean value of the second and third measurements was recorded as the final BP measurement.

After at least 8 h of fasting, blood and random mid-stream urine samples were obtained in the morning. After appropriate transport to the storage units at the Central Testing Institute, the samples were analyzed within 24 h for fasting blood glucose (FBG), high-density lipoprotein cholesterol (HDL-C), low-density lipoprotein cholesterol (LDL-C), total cholesterol (TC), and triglyceride (TG) levels, as well as white blood cell count (WBC), using a Hitachi automatic analyzer 7600 (Tokyo, Japan). The glycated hemoglobin (HbA1c) level was also measured by high-performance liquid chromatography (Tosoh HLC-723G7, Tokyo, Japan). Urine and serum creatinine levels and the urine albumin level were determined by kinetic colorimetry and turbidimetric assays using the Hitachi Automatic Analyzer 7600. We calculated the estimated glomerular filtration rate (eGFR) using the Chronic Kidney Disease Epidemiology Collaboration (CKD-EPI) equation: eGFR (mL/[min·1.73 m^2^])  = 141× min (serum creatinine/κ, 1)^α^ × max (serum creatinine/κ, 1)^−1.209^ × 0.993^Age^ × 1.018 [if female] × 1.159 [if African American], where κ is 0.7 for women and 0.9 for men, α is −0.411 for men and −0.329 for women, min indicates the minimum level of serum creatinine/κ or 1, and max indicates the maximum level of serum creatinine/κ or 1 [39].

### Definition of obesity and abdominal obesity groups

We calculated body mass index (BMI) as weight (kg) divided by the square of the height (m). Based on these results, we defined obesity as a BMI of ≥25 kg/m^2^ [40], [41]. The cut-off values for abdominal obesity were defined as a WC ≥90 cm for men and ≥80 cm for women [42]. We divided subjects into the following four groups: non-obese/normal WC group; non-obese/high WC group; obese/normal WC group; and obese/high WC group.

### Definition of HTN and controlled HTN groups

HTN was defined as the use of antihypertensive medications or being diagnosed by a doctor or having BP of ≥140/90 mmHg. The ‘HTN group’ was defined as those who had HTN, whereas the ‘non-HTN group’ as those who did not have HTN. The ‘HTN group’ was divided into the ‘controlled HTN group’ and ‘uncontrolled HTN group’. The ‘controlled HTN group’ included hypertensive subjects whose BP was <140/90 mmHg, whereas the ‘uncontrolled HTN group’ included hypertensive subjects whose BP was ≥140/90 mmHg [43].

### Definition of albuminuria

Normoalbuminuria was defined as a urine ACR <30 mg/g. Microalbuminuria was defined as 30≤ urine ACR <300 mg/g, and macroalbuminuria was defined as urine ACR ≥300 mg/g [7], [8].

### Statistical Analysis

Data are presented as either means ± standard errors (SEs) for continuous variables or as percentage (SE) for categorical variables. To analyze the baseline characteristics of the study participants, we divided our study subjects according to the presence of HTN and control of HTN, to compare the mean values of cardiometabolic risk factors; the chi-squared test was used for categorical variables and t-test was used for continuous variables. To compare the prevalence and control of HTN with the ACR tertile in obese and non-obese subjects, we used the chi-squared test. To identify the associations of SBP and DBP with ACR, we performed multiple regression analysis. Multiple logistic regression analyses were applied to examine the odds ratios (ORs) and 95% confidence intervals (CIs) of the prevalence and control of HTN according to the ACR among the subjects. Because the KNHANES included weights, statistical analysis was performed using the SAS (version 9.2; SAS Institute Inc., Cary, NC, USA) survey procedure to account for the complex sampling design and to provide approximations of the whole Korean population. We used logarithmic transformation for the variables with skewed distributions. All statistical tests were two-tailed and a p value of <0.05 was considered statistically significant.

## Results

In non-obese subjects, the HTN group was older and had a higher proportion of male subjects. The BMI, WC, ACR, SBP, DBP, WBC, FBG, HbA1c, TG, LDL-C, and TC levels were higher in the HTN group than the non-HTN group (all p values <0.01). However, the HDL-C level and eGFR were lower in the HTN group than in the non-HTN group (both p values <0.001). The HTN group had a higher proportion of individuals with heavy alcohol intake, lower income, and lower education, as well as higher prevalence of DM, as compared to the non-HTN group (all p values <0.001). Total energy intake and percentage of fat and protein intake were lower, but carbohydrate intake was higher in the HTN group than in the non-HTN group (all p values <0.001). Among obese subjects, the HTN group was older than non-HTN group. The BMI, WC, ACR, SBP, DBP, WBC, FBG, HbA1c, and TG levels were higher in the HTN group than the non-HTN group (all p values <0.05). However, eGFR, HDL-C, LDL-C, and TC levels were higher in the HTN group than in the non-HTN group (p value <0.001). The HTN group had a higher proportion of individuals with heavy alcohol intake, lower income, and lower education, as well as a higher prevalence of DM, as compared to the non-HTN group (all p values <0.001). The percentage of fat and protein intake were lower, but the carbohydrate intake was higher in the HTN group than in the non-HTN group (all p values <0.001) (Table 1).

**Table 1:** General characteristics of subjects with and without obesity and HTN.

Variable	Non-obese subjects		p value*	Obese subjects		p value*
	non-HTN	HTN		non-HTN	HTN	
Unweighted n	4627	1736		1680	1461	
Sex (male, %)	48.2(0.9)	53.4(1.5)	0.002	56.9(1.5)	57.8(1.6)	0.671
Age (yrs, mean ± SE)	40.8±0.3	58.9±0.5	<0.001	42.0±0.4	54.1±0.6	<0.001
BMI (kg/m^2^, mean ± SE)	21.7±0.0	22.5±0.1	<0.001	27.5±0.1	27.9±0.1	0.004
WC (cm, mean ± SE)	75.7±0.1	80.1±0.2	<0.001	90.2±0.3	92.4±0.3	<0.001
SBP (mmHg, mean ± SE)	110.4±0.2	135.4±0.6	<0.001	114.7±0.3	133.7±0.5	<0.001
DBP (mmHg, mean ± SE)	72.4±0.2	83.1±0.4	<0.001	76.0±0.3	85.2±0.4	<0.001
FBG (mg/dL, mean ± SE)	92.3±0.3	102.6±0.8	<0.001	98.5±0.7	105.5±0.7	<0.001
HbA1c (%, mean ± SE)	5.5±0.0	5.9±0.0	<0.001	5.7±0.0	6.0±0.0	<0.001
TG (mg/dL, mean ± SE)**	91.2±1.8	126.8±4.9	<0.001	135.5±5.0	153.7±5.9	<0.001
HDL-C (mg/dL, mean ± SE)	54.9±0.3	51.7±0.4	<0.001	48.1±0.3	47.9±0.4	<0.001
LDL-C (mg/dL, mean ± SE)	107.4±0.6	110.9±1.2	0.008	119.2±1.0	114.5±1.2	<0.001
TC (mg/dL, mean ± SE)	183.5±0.7	191.6±1.3	<0.001	198.0±1.2	196.6±1.3	<0.001
WBC (×10^3^/µL, mean ± SE)**	5.6±0.1	6.0±0.1	<0.001	6.2±0.1	6.3±0.1	0.033
eGFR (ml/min/1.73 m^2^, mean ± SE)	97.8±0.4	87.8±0.5	<0.001	95.4±0.5	88.0±0.7	<0.001
ACR (mg/g Cr, mean ± SE) **	3.8±0.1	8.4±0.7	<0.001	4.5±0.3	8.5±0.8	<0.001
Heavy alcohol intake (yes,%)	16.1(0.7)	22.6(1.5)	<0.001	20.9(1.4)	27.9(1.5)	<0.001
Current smoking (yes,%)	23.7(0.9)	22.7(1.4)	0.277	28.2(1.4)	24.7(1.6)	0.544
Regular exercise (yes,%)	18.4(0.8)	17.5(1.3)	0.101	22.3(1.3)	15.7(1.2)	0.563
Income (lowest quartile,%)	11.1(0.7)	27.2(1.4)	<0.001	10.3(1.0)	22.2(1.3)	<0.001
Education (≤9 yrs,%)	18.7(0.8)	56.7(1.6)	<0.001	22.6(1.3)	46.3(1.8)	<0.001
DM (yes, %)	3.7(0.3)	17.5(1.2)	<0.001	7.5(0.8)	18.6(1.3)	<0.001
HTN medication (yes, %)	.	52.7(1.6)	.	.	56.5(1.9)	.
Controlled HTN (yes, %)†		39.7(1.6)			39.5(1.8)	
Total energy intake (kcal, mean ± SE)	2098.3±21.5	1943.3±30.9	<0.001	2160.5±35.8	2104.2±43.2	0.302
Protein (%, mean ± SE)	20.1±0.2	15.2±0.3	<0.001	20.3±0.3	16.7±0.4	<0.001
Fat (%, mean ± SE)	15.0±0.1	14.2±0.2	<0.001	15.4±0.2	14.4±0.2	<0.001
Carbohydrate (%, mean ± SE)	64.9±0.3	70.5±0.4	<0.001	64.3±0.4	68.9±0.5	<0.001

Data are presented as mean ± standard error (SE) or percentages (SE).

*p values were obtained by the chi-Square test and t-test.

**Log transformation and data are presented as geometric mean ± standard error (SE).

† Controlled HTN means subjects who have BP <140/90 mmHg.

HTN, hypertension; BMI, body mass index; WC, waist circumference; SBP, systolic blood pressure; DBP, diastolic blood pressure; FBG, fasting blood glucose; TG, triglyceride; HDL-C, high density lipoprotein cholesterol; LDL-C, low density lipoprotein cholesterol; TC, total cholesterol; WBC, white blood cell; eGFR, estimated glomerular filtration rate; ACR, albumin creatinine ratio; DM, diabetes mellitus.

**Figure 1:**
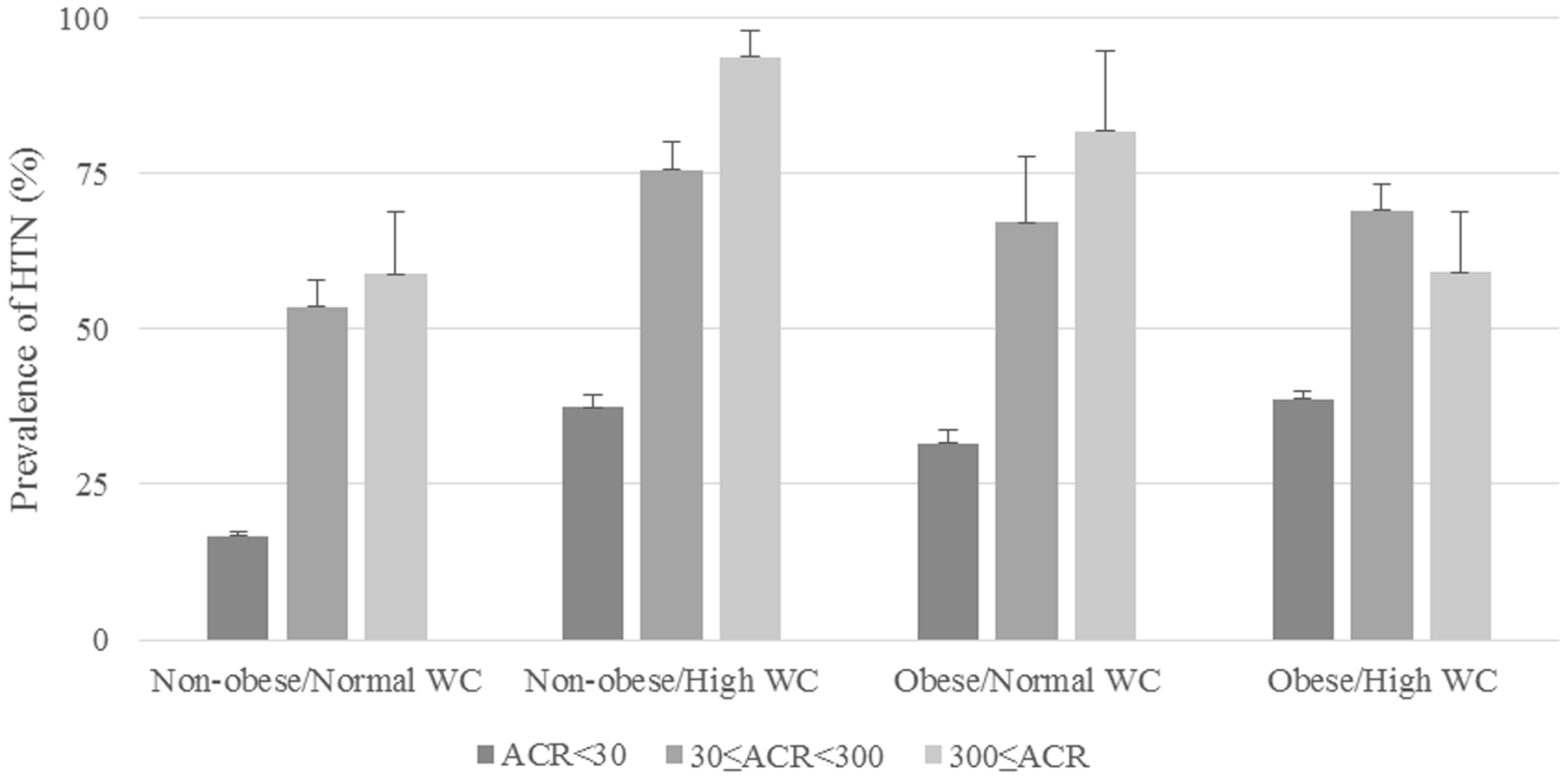
Prevalence of HTN in subjects with and without obesity and abdominal obesity by tertile of ACR. All p values for trends were <0.001. All p values were <0.001 between two groups; ACR <30 vs. 30≤ ACR <300, and ACR <30 vs. ACR ≥300 at all groups. HTN, hypertension; ACR, albumin–creatinine ratio; WC, waist circumference.

**Figure 2:**
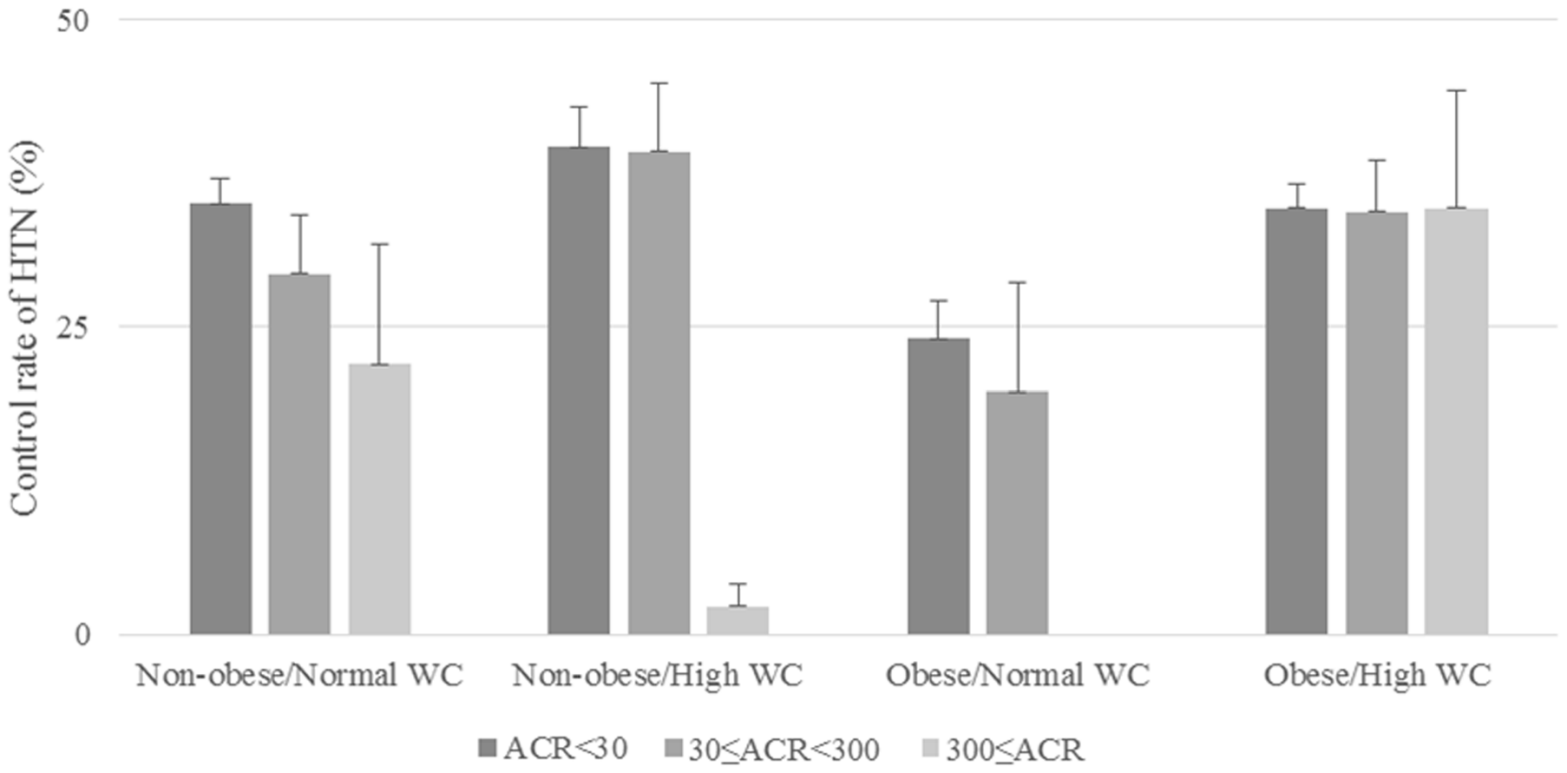
HTN control in subjects with and without obesity and abdominal obesity by tertile of ACR. P value for trend was <0.001 for the non-obese/high WC group only. P values were <0.05 between two groups; ACR <30 vs. 30≤ ACR <300, and ACR <30 vs. ACR ≥300 for the non-obese/normal WC group only. The p values and p values for trends were not statistically significant in the other three groups. HTN, hypertension; ACR, albumin–creatinine ratio; WC, waist circumference.

We compared the prevalence of HTN with the ACR tertiles in subjects with and without obesity and abdominal obesity (Figure 1). In all groups, the prevalence of HTN increased as ACR increased (all p values for trends were <0.001). There were differences in the prevalence of HTN between two groups among the four obese and non-obese groups when ACR was analyzed: ACR <30 vs. 30≤ ACR <300, and ACR <30 vs. ACR ≥300. In Figure 2, we compared the control rate of HTN according to the ACR tertile. In the non-obese/high WC group only, the rate of HTN control decreased significantly as the ACR increased (p value for trend <0.001). The control of HTN was significantly different between two groups: ACR <30 vs. 30≤ ACR <300, and ACR <30 vs. ACR ≥300 in non-obese/normal WC group (both p values <0.05). The p values and p values for trends were not statistically significant in the other three groups.

We analyzed the relationship between BP and ACR among the entire study population and hypertensive subjects according to the obesity status (Table 2). In general subjects, SBP and DBP were positively correlated with ACR in all groups (all p values <0.005). In hypertensive subjects, SBP was also positively correlated with ACR in all groups (all p values <0.01), but DBP was only positively correlated with ACR in non-obese/high WC group and obese/high WC group (p value  = 0.004 and 0.029, respectively).

**Table 2:** Correlation between blood pressure and ACR in subjects with and without obesity and abdominal obesity.

	Non-obese/Normal WC		Non-obese/High WC		Obese/Normal WC		Obese/High WC	
	beta	p value*	beta	p value*	beta	p value*	beta	p value*
General subjects								
SBP	2.37	<0.001	4.19	<0.001	3.06	<0.001	2.95	<0.001
DBP	0.62	0.002	1.80	<0.001	1.39	0.003	0.08	<0.001
Hypertensive subjects								
SBP	2.08	<0.001	2.83	<0.001	2.34	0.006	2.22	<0.001
DBP	0.47	0.127	1.13	0.004	0.84	0.092	0.65	0.029

*p value was obtained by age-adjusted multiple regression analysis.

ACR, albumin–creatinine ratio; WC, waist circumference; HTN, hypertension; SBP, systolic blood pressure; DBP, diastolic blood pressure.

The ORs for the prevalence and control of HTN with respect to the ACR are given in Table 3. The ORs of the reference groups (ACR <30 mg/g) were expressed as 1.00. In the non-obese/normal WC group, the ORs [95% CIs] for the prevalence of HTN were 3.20 [2.21–4.63] in microalbuminuria level and 3.09 [1.05–9.14] in macroalbuminuria level after adjusting for age, sex, body mass index, alcohol intake, smoking, exercise, income, education, and DM status. The ORs for the controlled HTN were lower than 1 in subjects with microalbuminuria and macroalbuminuria after adjusting for age, sex, body mass index, alcohol intake, smoking, exercise, income, education, DM status, and HTN medication (OR [95% CI]: 0.48 [0.26–0.89] and 0.25 [0.09–0.73], respectively). The obese/normal WC group were also likely to have HTN (OR [95% CI]: 3.10 [1.56–6.15] in microalbuminuria level and 21.75 [3.66–129.04] in macroalbuminuria level), but less likely to have HTN control in macroalbuminuria level (OR [95% CI]: 0.04 [0.01–0.15]).

**Table 3:** Adjusted odds ratios of HTN prevalence and control according to states of obesity.

ACR	Non-obese/Normal WC		Non-obese/High WC		Obese/Normal WC		Obese/High WC	
(mg/g)	Model 1	Model 2*	Model 1	Model 2*	Model 1	Model 2*	Model 1	Model 2*
HTN prevalence								
<30	1.00	1.00	1.00	1.00	1.00	1.00	1.00	1.00
30–300	3.09 (2.14–4.47)	3.20 (2.21–4.63)	2.40 (0.75–7.67)	1.90 (0.60–5.98)	3.61 (2.01–6.47)	3.10 (1.56–6.15)	2.53 (1.64–3.92)	2.63 (1.69–4.11)
≥300	3.32 (1.08–10.26)	3.09 (1.05–9.14)	3.09 (0.44–21.76)	2.02 (0.27–14.98)	20.14 (4.20–96.58)	21.75 (3.66–129.04)	1.30 (0.64–2.61)	1.16 (0.52–2.59)
Controlled HTN								
<30	1.00	1.00	1.00	1.00	1.00	1.00	1.00	1.00
30–300	0.61 (0.37–0.98)	0.48 (0.26–0.89)	0.35 (0.08–1.42)	0.24 (0.05–1.10)	1.10 (0.64–1.87)	0.71 (0.38–1.33)	0.84 (0.53–1.33)	0.89 (0.51–1.58)
≥300	0.55 (0.21–1.40)	0.25 (0.09–0.73)	1.27 (0.15–10.91)	1.44 (0.17–12.30)	0.23 (0.07–0.76)	0.04 (0.01–0.15)	0.81 (0.36–1.79)	0.51 (0.22–1.19)

Odds ratios and 95% confidence intervals were obtained by multiple logistic regression analysis.

Model 1 was adjusted for age, sex, and body mass index.

*Model 2 was adjusted for the covariates of model 1 plus alcohol intake, smoking, exercise, income, education, and diabetes mellitus status in the analysis of HTN prevalence. From the analysis of HTN control, HTN medication was added to the covariates of model 2.

HTN, hypertension; ACR, albumin–creatinine ratio; WC, waist circumference.

## Discussion

In this study, the non-obese and normal WC group showed an increased prevalence and decreased control of HTN in both microalbuminuria and macroalbuminuria levels. The OR for the prevalence of HTN was lower in obese and high WC group than in non-obese and normal WC group by microalbuminuria level. However, the obese and high WC group did not show a consistent relationship between HTN control and ACR.

In general, microalbuminuria is an effective biological marker for subjects with frequent cardiovascular events [44], and numerous studies have shown that microalbuminuria is associated with CVD and HTN [17], [19], [45]–[48]. Studies conducted in Korea also noted that microalbuminuria is positively correlated with BP, although these studies had limited applicability to the general population, as they only included subjects with type 2 DM or general subjects in a restricted population [27]–[29]. Endothelial dysfunction is one of the hypothetical mechanisms underlying the relationship between microalbuminuria and HTN [20]. Functional and structural remodeling of the arterial wall, which causes arterial stiffness, affects glomerular basement membranes and alters glomerular barrier permeability, which may result in albuminuria [46], [49]. Glomerular damage via the direct transmission of pulsatile stress to the glomeruli and an advanced arterial atherosclerotic process is another potential mechanism [22], [50], [51]. Therefore, it is possible to link the relationship between uncontrolled HTN and albuminuria. Poor control of HTN is particularly associated with elevated SBP, which is a more harmful risk factor than DBP for the heart, brain, and kidney [52]–[55]. This study also showed a positive correlation between ACR and BP.

Numerous studies have shown a relationship between ACR and obesity or abdominal obesity in diabetic, non-diabetic, or hypertensive subjects. However, some controversies exist regarding the relationship between ACR and obesity status [31]–[36], [56]. The Diabetes Control and Complications Trial [35] and the Look Action for Health in Diabetes study [36] showed a positive relationship between obesity status and ACR in Caucasian subjects with and without DM [34], [57], [58]. Such positive correlations were also found in South Asians [59] and South Koreans [33]. However, some studies found no relationship between obesity status and ACR [56], [60]–[62]. In this study, non-obese and normal WC subjects with albuminuria had more odds of having HTN and uncontrolled HTN than obese and high WC subjects. This result suggests that non-obese subjects and normal WC subjects may be more susceptible to the development of albuminuria due to high BP than obese and high WC subjects. This finding indicates that other mechanisms likely affect the relationship between ACR and HTN according to obesity status. Hypothetical mechanisms that support the positive correlation between ACR and obesity include decreased adiponectin levels [63], elevated insulin resistance [64], and structural alterations with glomerular dysfunction such as hyperperfusion and hyperfiltration [65]. But some structural or hormonal mechanisms might protect the kidney against the direct harmful effects of high BP and high glucose levels, as observed in the obesity paradox of CVD and end-stage renal disease [66]–[68]. One possible mechanism is that obese patients might have better tolerance to BP fluctuations than non-obese patients, as many obese subjects have higher SBP levels [69]. Other hypothetical mechanisms include the protective role of increased soluble tumor necrosis factor alpha (TNF-α) receptors through the neutralization of the harmful effects of TNF-α [70] and sequestration of uremic toxins by abundant adipose tissues [71]. Further studies are needed to evaluate the influence of obesity on albuminuria development in patients with HTN.

This study has some limitations. First, this was a cross-sectional study, and therefore, we could not show causal relationships. Second, only one morning urine sample was collected for evaluating albuminuria. In general, a morning urine sample after fasting is more concentrated than other urine samples collected at other time points; therefore, a false-positive result for albuminuria might have occurred. The mean values of ACRs measured from several urine samples would provide more precise results. Third, the definition of albuminuria is complicated by three factors: variable sampling techniques (including consequent accuracy of sampling errors), different albumin collection techniques, and diversity in albumin quantity ranges. Thus, some experimental errors could have been introduced by urine albumin analysis [39]. Fourth, we did not evaluate inflammatory markers such as C-reactive protein, TNF-α, and other cytokines such as leptin and adiponectin, which are involved in the mechanism of albuminuria.

Nevertheless, this study has strengths. It is the first large epidemiologic survey designed to determine the association between the prevalence and control of HTN and ACR in the overall South Korean population according to obesity status. Like other studies, the prevalence and control of HTN were associated with albuminuria; however, there were some unexpected results in non-obese and non-abdominal obese subjects, which require further evaluation.

In conclusion, we found that non-obese and normal WC group were more likely to have HTN, but less likely to have HTN control in both microalbuminuria and macroalbuminuria levels. It is therefore important to monitor BP in individuals with albuminuria even if they are not obese and have a normal WC. Further studies are needed to evaluate the mechanisms and relationship between ACR and HTN in subjects according to obesity status.

## References

[1] Deaton C, Froelicher ES, Wu LH, Ho C, Shishani K, et al (2011) The global burden of cardiovascular disease. J Cardiovasc Nurs 26: S5–14. 21659814 10.1097/JCN.0b013e318213efcf

[2] Chobanian AV, Bakris GL, Black HR, Cushman WC, Green LA, et al (2003) Seventh report of the Joint National Committee on Prevention, Detection, Evaluation, and Treatment of High Blood Pressure. Hypertension 42: 1206–1252. 14656957 10.1161/01.HYP.0000107251.49515.c2

[3] Lawes CM, Rodgers A, Bennett DA, Parag V, Suh I, et al (2003) Blood pressure and cardiovascular disease in the Asia Pacific region. J Hypertens 21: 707–716. 12658016 10.1097/00004872-200304000-00013

[4] Wachtell K, Ibsen H, Olsen MH, Borch-Johnsen K, Lindholm LH, et al (2003) Albuminuria and cardiovascular risk in hypertensive patients with left ventricular hypertrophy: the LIFE study. Ann Intern Med 139: 901–906. 14644892 10.7326/0003-4819-139-11-200312020-00008

[5] Roest M, Banga JD, Janssen WM, Grobbee DE, Sixma JJ, et al (2001) Excessive urinary albumin levels are associated with future cardiovascular mortality in postmenopausal women. Circulation 103: 3057–3061. 11425768 10.1161/hc2501.091353

[6] Arnlov J, Evans JC, Meigs JB, Wang TJ, Fox CS, et al (2005) Low-grade albuminuria and incidence of cardiovascular disease events in nonhypertensive and nondiabetic individuals: the Framingham Heart Study. Circulation 112: 969–975. 16087792 10.1161/CIRCULATIONAHA.105.538132

[7] Mogensen CE, Chachati A, Christensen CK, Close CF, Deckert T, et al (1985) Microalbuminuria: an early marker of renal involvement in diabetes. Uremia Invest 9: 85–95. 3915933 10.3109/08860228509088195

[8] Eknoyan G, Hostetter T, Bakris GL, Hebert L, Levey AS, et al (2003) Proteinuria and other markers of chronic kidney disease: a position statement of the national kidney foundation (NKF) and the national institute of diabetes and digestive and kidney diseases (NIDDK). Am J Kidney Dis 42: 617–622. 14520612 10.1016/s0272-6386(03)00826-6

[9] Jager A, Kostense PJ, Ruhe HG, Heine RJ, Nijpels G, et al (1999) Microalbuminuria and peripheral arterial disease are independent predictors of cardiovascular and all-cause mortality, especially among hypertensive subjects: five-year follow-up of the Hoorn Study. Arterioscler Thromb Vasc Biol 19: 617–624. 10073965 10.1161/01.atv.19.3.617

[10] Klausen K, Borch-Johnsen K, Feldt-Rasmussen B, Jensen G, Clausen P, et al (2004) Very low levels of microalbuminuria are associated with increased risk of coronary heart disease and death independently of renal function, hypertension, and diabetes. Circulation 110: 32–35. 15210602 10.1161/01.CIR.0000133312.96477.48

[11] Romundstad S, Holmen J, Kvenild K, Hallan H, Ellekjaer H (2003) Microalbuminuria and all-cause mortality in 2,089 apparently healthy individuals: a 4.4-year follow-up study. The Nord-Trondelag Health Study (HUNT), Norway. Am J Kidney Dis 42: 466–473. 12955674 10.1016/s0272-6386(03)00742-x

[12] Weir MR (2004) Microalbuminuria in type 2 diabetics: an important, overlooked cardiovascular risk factor. J Clin Hypertens (Greenwich) 6: 133–142 quiz – 10.1111/j.1524-6175.2004.02524.x PMC8109345 15010646

[13] Yuyun MF, Khaw KT, Luben R, Welch A, Bingham S, et al (2004) Microalbuminuria independently predicts all-cause and cardiovascular mortality in a British population: The European Prospective Investigation into Cancer in Norfolk (EPIC-Norfolk) population study. Int J Epidemiol 33: 189–198. 15075168 10.1093/ije/dyh008

[14] Flack JM, Duncan K, Ohmit SE, Quah R, Liu X, et al (2007) Influence of albuminuria and glomerular filtration rate on blood pressure response to antihypertensive drug therapy. Vasc Health Risk Manag 3: 1029–1037. 18200821 PMC2350134

[15] Liu X, Wang K, Wang L, Tsilimingras D (2013) Microalbuminuria, macroalbuminuria and uncontrolled blood pressure among diagnosed hypertensive patients: the aspect of racial disparity in the NHANES study. Hypertens Res 36: 1100–1106. 23945963 10.1038/hr.2013.87

[16] Ong KL, Tso AW, Lam KS, Cheung BM (2008) Gender difference in blood pressure control and cardiovascular risk factors in Americans with diagnosed hypertension. Hypertension 51: 1142–1148. 18259031 10.1161/HYPERTENSIONAHA.107.105205

[17] Bianchi S, Bigazzi R, Campese VM (1999) Microalbuminuria in essential hypertension: significance, pathophysiology, and therapeutic implications. Am J Kidney Dis 34: 973–995. 10585306 10.1016/S0272-6386(99)70002-8

[18] Knight EL, Kramer HM, Curhan GC (2003) High-normal blood pressure and microalbuminuria. Am J Kidney Dis 41: 588–595. 12612982 10.1053/ajkd.2003.50120

[19] Tanaka S, Takase H, Dohi Y, Kimura G (2013) The prevalence and characteristics of microalbuminuria in the general population: a cross-sectional study. BMC Res Notes 6: 256. 23830507 10.1186/1756-0500-6-256 PMC3846492

[20] Deckert T, Feldt-Rasmussen B, Borch-Johnsen K, Jensen T, Kofoed-Enevoldsen A (1989) Albuminuria reflects widespread vascular damage. The Steno hypothesis. Diabetologia 32: 219–226. 2668076 10.1007/BF00285287

[21] Williams SA, Boolell M, MacGregor GA, Smaje LH, Wasserman SM, et al (1990) Capillary hypertension and abnormal pressure dynamics in patients with essential hypertension. Clin Sci (Lond) 79: 5–8. 2167790 10.1042/cs0790005

[22] Deen WM (2004) What determines glomerular capillary permeability? J Clin Invest 114: 1412–1414. 15545991 10.1172/JCI23577 PMC525751

[23] Kidney Disease Outcomes Quality I (2004) K/DOQI clinical practice guidelines on hypertension and antihypertensive agents in chronic kidney disease. Am J Kidney Dis 43: S1–290. 15114537

[24] Choi HJ (2012) Blood pressure variability and its management in hypertensive patients. Korean J Fam Med 33: 330–335. 23267418 10.4082/kjfm.2012.33.6.330 PMC3526715

[25] Assmann G, Schulte H (1989) Diabetes mellitus and hypertension in the elderly: concomitant hyperlipidemia and coronary heart disease risk. Am J Cardiol 63: 33H–37H. 10.1016/0002-9149(89)90113-6 2650522

[26] Tight blood pressure control and risk of macrovascular and microvascular complications in type 2 diabetes: UKPDS 38. UK Prospective Diabetes Study Group. BMJ 317: 703–713. PMC28659 9732337

[27] Kim BJ, Lee HJ, Sung KC, Kim BS, Kang JH, et al (2007) Comparison of microalbuminuria in 2 blood pressure categories of prehypertensive subjects. Circ J 71: 1283–1287. 17652896 10.1253/circj.71.1283

[28] Kim BJ, Lee HA, Kim NH, Kim MW, Kim BS, et al (2011) The association of albuminuria, arterial stiffness, and blood pressure status in nondiabetic, nonhypertensive individuals. J Hypertens 29: 2091–2098. 21881520 10.1097/HJH.0b013e32834b5627

[29] Kim YS, Kim HS, Oh HY, Lee MK, Kim CH, et al (2013) Prevalence of microalbuminuria and associated risk factors among adult Korean hypertensive patients in a primary care setting. Hypertens Res 36: 807–823. 23698804 10.1038/hr.2013.44

[30] Shin DI, Seung KB, Yoon HE, Hwang BH, Seo SM, et al (2013) Microalbuminuria is independently associated with arterial stiffness and vascular inflammation but not with carotid intima-media thickness in patients with newly diagnosed type 2 diabetes or essential hypertension. J Korean Med Sci 28: 252–260. 23400641 10.3346/jkms.2013.28.2.252 PMC3565137

[31] Chang A, Van Horn L, Jacobs DR, Liu K, Muntner P, et al (2013) Lifestyle-Related Factors, Obesity, and Incident Microalbuminuria: The CARDIA (Coronary Artery Risk Development in Young Adults) Study. American Journal of Kidney Diseases 62: 267. 23601954 10.1053/j.ajkd.2013.02.363 PMC3720776

[32] Thoenes M, Reil JC, Khan BV, Bramlage P, Volpe M, et al (2009) Abdominal obesity is associated with microalbuminuria and an elevated cardiovascular risk profile in patients with hypertension. Vasc Health Risk Manag 5: 577–585. 19649308 10.2147/vhrm.s5207 PMC2710972

[33] Nam GE, Han K, Park YG, Kim YH, Lee KS, et al (2013) Abdominal Obesity Is Associated with Albuminuria in Women: The 2011 Korea National Health and Nutrition Examination Survey. J Womens Health (Larchmt).. 10.1089/jwh.2013.4497 24286240

[34] Bonnet F, Marre M, Halimi JM, Stengel B, Lange C, et al (2006) Waist circumference and the metabolic syndrome predict the development of elevated albuminuria in non-diabetic subjects: the DESIR Study. J Hypertens 24: 1157–1163. 16685216 10.1097/01.hjh.0000226206.03560.ac

[35] de Boer IH, Sibley SD, Kestenbaum B, Sampson JN, Young B, et al (2007) Central obesity, incident microalbuminuria, and change in creatinine clearance in the epidemiology of diabetes interventions and complications study. J Am Soc Nephrol 18: 235–243. 17151331 10.1681/ASN.2006040394 PMC2622719

[36] Kramer H, Reboussin D, Bertoni AG, Marcovina S, Lipkin E, et al (2009) Obesity and albuminuria among adults with type 2 diabetes: the Look AHEAD (Action for Health in Diabetes) Study. Diabetes Care 32: 851–853. 19196893 10.2337/dc08-2059 PMC2671132

[37] Park HA (2013) The Korea national health and nutrition examination survey as a primary data source. Korean J Fam Med 34: 79. 23560205 10.4082/kjfm.2013.34.2.79 PMC3611106

[38] Hagstromer M, Oja P, Sjostrom M (2006) The International Physical Activity Questionnaire (IPAQ): a study of concurrent and construct validity. Public Health Nutr 9: 755–762. 16925881 10.1079/phn2005898

[39] Levey AS, Eckardt KU, Tsukamoto Y, Levin A, Coresh J, et al (2005) Definition and classification of chronic kidney disease: a position statement from Kidney Disease: Improving Global Outcomes (KDIGO). Kidney Int 67: 2089–2100. 15882252 10.1111/j.1523-1755.2005.00365.x

[40] Oh SW, Shin SA, Yun YH, Yoo T, Huh BY (2004) Cut-off point of BMI and obesity-related comorbidities and mortality in middle-aged Koreans. Obes Res 12: 2031–2040. 15687405 10.1038/oby.2004.254

[41] Weisell RC (2002) Body mass index as an indicator of obesity. Asia Pac J Clin Nutr 11 Suppl 8: S681–684.

[42] Grundy SM, Cleeman JI, Daniels SR, Donato KA, Eckel RH, et al (2005) Diagnosis and management of the metabolic syndrome: an American Heart Association/National Heart, Lung, and Blood Institute Scientific Statement. Circulation 112: 2735–2752. 16157765 10.1161/CIRCULATIONAHA.105.169404

[43] James PA, Oparil S, Carter BL, Cushman WC, Dennison-Himmelfarb C, et al (2014) 2014 evidence-based guideline for the management of high blood pressure in adults: report from the panel members appointed to the Eighth Joint National Committee (JNC 8). JAMA 311: 507–520. 24352797 10.1001/jama.2013.284427

[44] Yuyun MF, Adler AI, Wareham NJ (2005) What is the evidence that microalbuminuria is a predictor of cardiovascular disease events? Curr Opin Nephrol Hypertens 14: 271–276. 15821422 10.1097/01.mnh.0000165895.90748.3b

[45] Chico A, Tomas A, Novials A (2005) Silent myocardial ischemia is associated with autonomic neuropathy and other cardiovascular risk factors in type 1 and type 2 diabetic subjects, especially in those with microalbuminuria. Endocrine 27: 213–217. 16230776 10.1385/ENDO:27:3:213

[46] Stehouwer CD, Smulders YM (2006) Microalbuminuria and risk for cardiovascular disease: Analysis of potential mechanisms. J Am Soc Nephrol 17: 2106–2111. 16825333 10.1681/ASN.2005121288

[47] Cerasola G, Cottone S, Mule G (2010) The progressive pathway of microalbuminuria: from early marker of renal damage to strong cardiovascular risk predictor. J Hypertens 28: 2357–2369. 20842046 10.1097/HJH.0b013e32833ec377

[48] Jensen JS, Feldt-Rasmussen B, Strandgaard S, Schroll M, Borch-Johnsen K (2000) Arterial hypertension, microalbuminuria, and risk of ischemic heart disease. Hypertension 35: 898–903. 10775558 10.1161/01.hyp.35.4.898

[49] Weir MR (2007) Microalbuminuria and cardiovascular disease. Clin J Am Soc Nephrol 2: 581–590. 17699466 10.2215/CJN.03190906

[50] O'Rourke MF, Safar ME (2005) Relationship between aortic stiffening and microvascular disease in brain and kidney: cause and logic of therapy. Hypertension 46: 200–204. 15911742 10.1161/01.HYP.0000168052.00426.65

[51] Smulyan H, Safar ME (1997) Systolic blood pressure revisited. J Am Coll Cardiol 29: 1407–1413. 9180097 10.1016/s0735-1097(97)00081-8

[52] Chobanian AV (2007) Clinical practice. Isolated systolic hypertension in the elderly. N Engl J Med 357: 789–796. 17715411 10.1056/NEJMcp071137

[53] Cirillo M, Stellato D, Laurenzi M, Panarelli W, Zanchetti A, et al (2000) Pulse pressure and isolated systolic hypertension: association with microalbuminuria. The GUBBIO Study Collaborative Research Group. Kidney Int 58: 1211–1218. 10972683 10.1046/j.1523-1755.2000.00276.x

[54] Ovbiagele B, Diener HC, Yusuf S, Martin RH, Cotton D, et al (2011) Level of systolic blood pressure within the normal range and risk of recurrent stroke. JAMA 306: 2137–2144. 22089721 10.1001/jama.2011.1650

[55] Pini R, Cavallini MC, Bencini F, Silvestrini G, Tonon E, et al (2002) Cardiovascular remodeling is greater in isolated systolic hypertension than in diastolic hypertension in older adults: the Insufficienza Cardiaca negli Anziani Residenti (ICARE) a Dicomano Study. J Am Coll Cardiol 40: 1283–1289. 12383576 10.1016/s0735-1097(02)02159-9

[56] Rutkowski B, Czarniak P, Krol E, Szczesniak P, Zdrojewski T (2013) Overweight, obesity, hypertension and albuminuria in Polish adolescents–results of the Sopkard 15 study. Nephrol Dial Transplant 28 Suppl 4: iv204–211. 24068778 10.1093/ndt/gft328

[57] Pinto-Sietsma SJ, Navis G, Janssen WM, de Zeeuw D, Gans RO, et al (2003) A central body fat distribution is related to renal function impairment, even in lean subjects. Am J Kidney Dis 41: 733–741. 12666059 10.1016/s0272-6386(03)00020-9

[58] Hillege HL, Janssen WM, Bak AA, Diercks GF, Grobbee DE, et al (2001) Microalbuminuria is common, also in a nondiabetic, nonhypertensive population, and an independent indicator of cardiovascular risk factors and cardiovascular morbidity. J Intern Med 249: 519–526. 11422658 10.1046/j.1365-2796.2001.00833.x

[59] Chandie Shaw PK, Berger SP, Mallat M, Frolich M, Dekker FW, et al (2007) Central obesity is an independent risk factor for albuminuria in nondiabetic South Asian subjects. Diabetes Care 30: 1840–1844. 17456841 10.2337/dc07-0028

[60] Hoffmann IS, Jimenez E, Cubeddu LX (2001) Urinary albumin excretion in lean, overweight and obese glucose tolerant individuals: its relationship with dyslipidaemia, hyperinsulinaemia and blood pressure. J Hum Hypertens 15: 407–412. 11439316 10.1038/sj.jhh.1001193

[61] Lin WY, Pi-Sunyer FX, Liu CS, Li CI, Davidson LE, et al (2012) Central obesity and albuminuria: both cross-sectional and longitudinal studies in Chinese. PLoS One 7: e47960. 23251329 10.1371/journal.pone.0047960 PMC3520991

[62] Won JC, Lee YJ, Kim JM, Han SY, Noh JH, et al (2013) Prevalence of and factors associated with albuminuria in the Korean adult population: the 2011 Korea National Health and Nutrition Examination Survey. PLoS One 8: e83273. 24386169 10.1371/journal.pone.0083273 PMC3873941

[63] Schalkwijk CG, Chaturvedi N, Schram MT, Fuller JH, Stehouwer CD, et al (2006) Adiponectin is inversely associated with renal function in type 1 diabetic patients. J Clin Endocrinol Metab 91: 129–135. 16219717 10.1210/jc.2005-1117

[64] Bagby SP (2004) Obesity-initiated metabolic syndrome and the kidney: a recipe for chronic kidney disease? J Am Soc Nephrol 15: 2775–2791. 15504931 10.1097/01.ASN.0000141965.28037.EE

[65] Amann K, Benz K (2013) Structural renal changes in obesity and diabetes. Semin Nephrol 33: 23–33. 23374891 10.1016/j.semnephrol.2012.12.003

[66] Park J, Ahmadi SF, Streja E, Molnar MZ, Flegal KM, et al (2014) Obesity paradox in end-stage kidney disease patients. Prog Cardiovasc Dis 56: 415–425. 24438733 10.1016/j.pcad.2013.10.005 PMC4733536

[67] Gupta NK, de Lemos JA, Ayers CR, Abdullah SM, McGuire DK, et al (2012) The relationship between C-reactive protein and atherosclerosis differs on the basis of body mass index: the Dallas Heart Study. J Am Coll Cardiol 60: 1148–1155. 22939555 10.1016/j.jacc.2012.04.050

[68] McDonald SP, Collins JF, Johnson DW (2003) Obesity is associated with worse peritoneal dialysis outcomes in the Australia and New Zealand patient populations. J Am Soc Nephrol 14: 2894–2901. 14569099 10.1097/01.asn.0000091587.55159.5f

[69] Horwich TB, Fonarow GC, Hamilton MA, MacLellan WR, Woo MA, et al (2001) The relationship between obesity and mortality in patients with heart failure. J Am Coll Cardiol 38: 789–795. 11527635 10.1016/s0735-1097(01)01448-6

[70] Mohamed-Ali V, Goodrick S, Bulmer K, Holly JM, Yudkin JS, et al (1999) Production of soluble tumor necrosis factor receptors by human subcutaneous adipose tissue in vivo. Am J Physiol 277: E971–975. 10600783 10.1152/ajpendo.1999.277.6.E971

[71] Niebauer J, Volk HD, Kemp M, Dominguez M, Schumann RR, et al (1999) Endotoxin and immune activation in chronic heart failure: a prospective cohort study. Lancet 353: 1838–1842. 10359409 10.1016/S0140-6736(98)09286-1

